# Small-Data Deep Learning for Alzheimer-Spectrum Classification from Structural MRI: A Feasibility Study Using OASIS

**DOI:** 10.3390/jimaging12080352

**Published:** 2026-08-03

**Authors:** Ian D. Li, Choong-Yong Ung, Cristina Correia

**Affiliations:** Department of Molecular Pharmacology and Experimental Therapeutics, Mayo Clinic College of Medicine and Science, Rochester, MN 55905, USA; ianbubu2008@gmail.com (I.D.L.); ung.choongyong@mayo.edu (C.-Y.U.)

**Keywords:** Alzheimer’s disease, cognitive impairment, magnetic resonance imaging, artificial intelligence, convolutional neural network

## Abstract

Accurate estimation of Alzheimer’s disease (AD) severity from structural magnetic resonance imaging (MRI) remains difficult, as disease-associated anatomical alterations are often subtle and publicly available datasets are typically too small to support robust deep learning model training. This feasibility study sought to determine how much Alzheimer’s disease spectrum-related information could be extracted from a small structural MRI cohort using a deliberately lightweight two-dimensional convolutional neural network (2D CNN), and whether transfer learning improves model performance. This study was intended as a methodological proof of concept rather than the development of a clinically deployable diagnostic tool. Structural scans and Clinical Dementia Rating (CDR) labels from the OASIS-1 dataset were filtered to 214 subjects: 124 cognitively normal (CN), 65 with mild cognitive impairment (MCI; CDR = 0.5), and 25 with AD-level impairment (CDR ≥ 1). A compact 2D CNN trained from scratch and a transfer learning model (frozen ImageNet MobileNetV2 features) were evaluated on four binary tasks (CN vs. AD, MCI vs. AD, CN vs. MCI, and CN vs. any impairment) under identical pre-processing and subject-level repeated 5-fold cross-validation (10 repeats), with the decision threshold tuned only on an inner split. Discrimination was summarized by ROC-AUC with 95% confidence intervals (CIs), permutation tests against chance, and per-task sensitivity and specificity. The from-scratch CNN recovered only a broad normal-versus-impaired signal (CN vs. any impairment AUC 0.59) and was at chance on adjacent-stage tasks (MCI vs. AD 0.41; CN vs. MCI 0.51). Transfer learning improved every task: CN vs. AD AUC 0.745 (95% CI 0.730–0.763), CN vs. any impairment 0.642, CN vs. MCI 0.601, and MCI vs. AD 0.599. On an independent OASIS-2 cohort, the transfer learning CN vs. AD model retained AUC 0.748. In this small-data regime, transfer learning recovers substantially more Alzheimer-spectrum signals than a from-scratch CNN, but performance remains modest because it is bounded by CDR-based, non-biomarker-confirmed labels, suggesting the model separates CDR-defined cognitive-status groups rather than detecting AD pathology.

## 1. Introduction

Alzheimer’s disease (AD) is a progressive neurodegenerative disorder in which structural brain changes accumulate years before overt dementia [1,2,3]. Structural magnetic resonance imaging (MRI) is therefore an appealing tool for an Alzheimer-spectrum assessment because it is noninvasive, widely available, and sensitive to neurodegeneration in vulnerable regions such as the medial temporal lobe and association cortex. At the same time, the earliest MRI abnormalities are subtle, spatially diffused, and partly overlap with normal aging and other causes of cognitive impairment. This makes early classification from MRI alone difficult, especially in individuals at the boundary between cognitively normal aging and mild impairment [4,5].

Deep learning has intensified interest in MRI-based Alzheimer’s research because convolutional neural networks (CNNs) can learn distributed image features that may not be captured by manually defined regions of interest. However, much of the field’s best-known evidence has been built on large research datasets and computationally intensive pipelines. For example, Liu et al. developed and externally validated a 3D deep learning model on the Alzheimer’s Disease Neuroimaging Initiative (ADNI) and National Alzheimer’s Coordinating Center (NACC) but still found broader normal-versus-impaired discrimination easier than the more clinically challenging problem of detecting mild cognitive impairment, while also showing that performance improved as training-set size increased [6]. In parallel, recent reviews of medical-imaging AI have emphasized that limited annotated data remain one of the central barriers to deep learning, motivating the growing interest in data-efficient and few-shot approaches [7]. Together, these studies suggest that a key open question is how many clinically relevant signals can be recovered by deep learning from small structural MRI datasets.

Recent work on data-efficient neuroimaging deep learning has identified limited labeled data as the primary bottleneck, despite advances in self-supervised pretraining, transfer learning, and imbalance-aware training for structural MRI. This challenge directly motivates the small-data feasibility question investigated here [8,9,10,11,12].

The present study addresses small-data questions using the open access OASIS-1 cohort introduced by Marcus et al., a public cross-sectional structural MRI resource that includes older adults with and without clinically defined Alzheimer-associated cognitive impairment [13]. Using a filtered subset of 214 subjects from OASIS-1, a lightweight slice-based 2D convolutional neural network (2D CNN) was evaluated with subject-level repeated 5-fold cross-validation across binary Clinical Dementia Rating (CDR)-defined classification tasks. Framed this way, the goal of this study is not to claim a definitive stand-alone diagnostic tool, but to test the feasibility and limits of small-scale MRI deep learning: whether a compact model trained on a modest public dataset can detect a meaningful Alzheimer-spectrum signal, and where structural MRI remains too ambiguous to support confident early classification on its own. The contribution of this work is methodological rather than architectural. First, it provides a transparent evaluation framework that minimizes bias by preventing subject-level information leakage, allowing us to determine how much Alzheimer’s disease spectrum-related information can be recovered by a minimal 2D CNN and a frozen-feature transfer learning model from a small public MRI cohort across four clinically defined classification tasks.

Secondly, it introduces a classifier-derived similarity analysis to quantify how similar the diagnostic groups appeared to the classifier. This analysis generated a classifier-derived visualization of class separability, revealing which disease categories (CN, MCI, and AD) exhibited the greatest overlap in the learned representations and were therefore most frequently confused.

Throughout, classes are defined strictly by CDR: cognitively normal (CN, CDR = 0), mild/early impairment operationalized as MCI (CDR = 0.5), and Alzheimer-level impairment (AD, CDR ≥ 1). One framing point applies throughout: OASIS-1 labels are CDR-based clinical ratings, not amyloid or tau biomarker confirmations. The models are therefore trained to separate CDR-defined cognitive-status groups and cannot be interpreted as detecting Alzheimer’s pathology itself.

## 2. Materials and Methods

### 2.1. Materials

This study used the publicly available Open Access Series of Imaging Studies (OASIS-1) cross-sectional dataset [13], which contains de-identified structural brain MRI scans and associated clinical information from older adults across a range of cognitive states. The primary study materials were the structural individual T1-weighted MRI scans and volume for each subject and the corresponding Clinical Dementia Rating (CDR) score used for image classification. Image processing, model development, and performance evaluation were conducted in Python (v3.10) within the Google Colab environment. TensorFlow (v2.15) and Keras were used to build the convolutional neural network. NumPy (v1.25) and Pandas (v2.0) supported numerical computing and metadata handling, NiBabel (v5.2; https://github.com/nipy/nibabel/releases, accessed on 6 March 2026) was used to load NIfTI-format MRI volumes, Matplotlib (v3.7) was used for image inspection and visualization, and Scikit-learn (v1.3) was used for subject-level data splitting, stratified cross-validation, and confusion matrix computation. The pathlib standard library module was used for file system navigation. A complete listing of all software packages and their versions is provided in Supplementary Table S1. The overall workflow was intentionally kept lightweight so that the project could evaluate whether a compact deep learning pipeline could learn clinically relevant Alzheimer-spectrum signals from a relatively small public MRI cohort.

### 2.2. Procedures

The analytic workflow included cohort definition, image pre-processing, 2D CNN development, and subject-level model evaluation.

#### 2.2.1. Data Collection

Structural MRI volumes and accompanying clinical data were obtained from the OASIS-1 cross-sectional release [13]. The OASIS-1 dataset contains 416 subjects aged 18–96 years, each with 3 or 4 T1-weighted structural MRI scans. Clinical Dementia Rating (CDR) is a 5-point scale used to stage the severity of dementia, particularly in Alzheimer’s disease, where a CDR value of 0.0 corresponds to healthy individuals, 0.5 corresponds to mild cognitive individuals, and higher values correspond to Alzheimer’s disease. Subjects without a valid CDR label were excluded before model training, reducing the cohort from 416 to 214 subjects. The excluded subjects were primarily younger adults (aged 18–59 years) who did not receive a CDR assessment as part of the original study protocol, as CDR scoring was performed only for subjects aged 60 years and older. After filtering, the final analytic cohort included 214 subjects, with each subject represented by one three-dimensional MRI volume and one clinical label. For modeling purposes, subjects were grouped according to CDR score into cognitively normal (CN; CDR = 0), mild cognitive impairment/early impairment (MCI; CDR = 0.5), and Alzheimer-level impairment (AD; CDR ≥ 1). Note that CDR = 0.5 was operationalized as MCI for this analysis. The final class distribution was 124 CN subjects, 65 MCI subjects, and 25 AD subjects. Because this study aimed to determine how well deep learning performs in a limited-data setting, classification was formulated as a set of binary tasks rather than a single multi-class problem. Four pairwise analyses were defined: CN vs. AD, MCI vs. AD, CN vs. MCI, and CN vs. any impairment (MCI + AD). This design allowed the model to test separability at multiple clinically relevant boundaries, including both broad normal-versus-impaired discrimination and the more difficult distinction between adjacent stages. Subjects with a CDR ≥ 1 (mild, moderate, and severe dementia) were collapsed into a single AD class because the number of subjects within each severity group was insufficient for stable subject-level modeling (25 AD subjects in total). The AD class should therefore be read as CDR ≥ 1 impairment rather than a homogeneous severity group.

We framed the problem as four independent binary tasks rather than one multi-class model. With only 25 AD subjects, a single three-way classifier would be dominated by the CN majority and would blur the specific boundaries of interest; independent binary tasks isolate separability at each clinically relevant transition (CN–MCI, MCI–AD, CN–AD, and normal-versus-any impairment) and feed directly into the pairwise similarity analysis.

#### 2.2.2. MRI Pre-Processing

Each subject’s T1-weighted MRI volume was stored in NIfTI-compressed format (.npz) and loaded using NiBabel (https://github.com/nipy/nibabel, accessed on 6 March 2026). Volumes were represented as three-dimensional NumPy arrays with voxel dimensions of approximately 256 × 256 × Z, where Z varied by scan. MRI volumes were converted from three-dimensional brain volumes into two-dimensional axial slices suitable for a slice-based CNN. A two-dimensional representation was chosen deliberately to reduce model complexity and computational burden in the setting of a modest sample size. For each subject, seven axial slices (7 × 214 = 1498 total slices across the cohort) near the center of the brain volume were selected by computing the midpoint of the axial dimension (z_mid = Z//2) and extracting slices at offsets of [−6, −4, −2, 0, +2, +4, +6] relative to z_mid. These central axial levels were chosen because they preserved substantial cortical and subcortical anatomy, including the hippocampus, entorhinal cortex, lateral ventricles, and temporal lobe structures that are among the earliest regions affected by Alzheimer-related neurodegeneration while avoiding the least informative superior and inferior extremes of the scan. These slices were used as input in the 2D CNN. Using multiple central slices also allowed the model to sample disease-relevant structural variation from each brain while maintaining a simple and reproducible pre-processing workflow. Additional pre-processing utilized NumPy for array manipulation, nilearn (version 0.10) for neuroimaging utilities, and Scikit-learn for data splitting.

**Figure 1:**
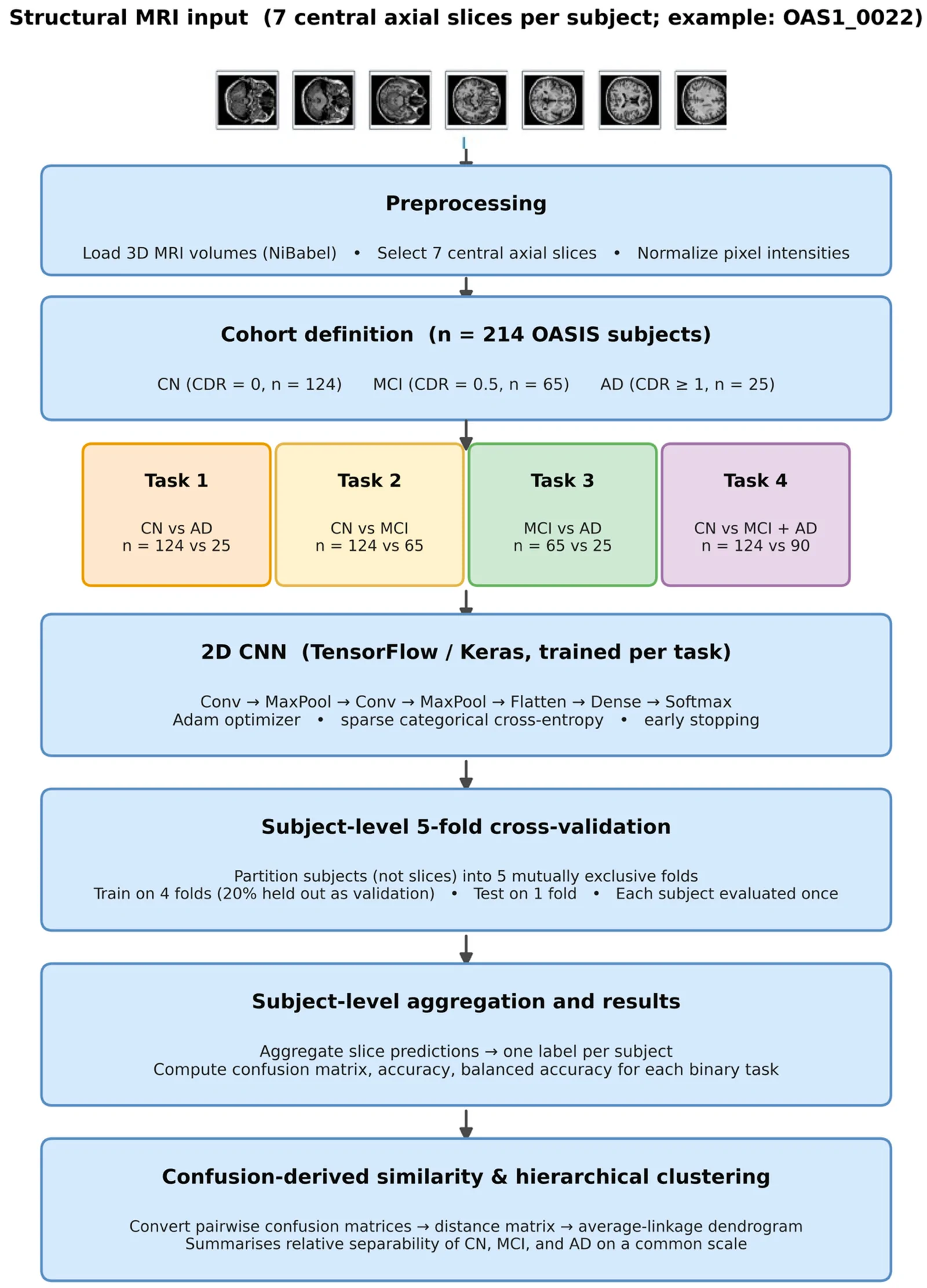
Analytic pipeline for the OASIS-1 small-data Alzheimer-spectrum classification study. Structural MRI volumes from 214 OASIS-1 subjects were loaded with NiBabel, and seven central axial slices per subject were selected and intensity-normalized. Subjects were grouped by Clinical Dementia Rating (CDR) into cognitively normal (CN; CDR = 0; *n* = 124), mild cognitive impairment (MCI; CDR = 0.5; *n* = 65), and Alzheimer-level impairment (AD; CDR ≥ 1; *n* = 25), and four binary classification tasks were defined. For each task, a lightweight 2D convolutional neural network was trained in TensorFlow/Keras (v2.15) using an Adam optimizer, sparse categorical cross-entropy loss, and early stopping. Performance was evaluated with subject-level 5-fold cross-validation.

Before model input, pixel intensities were normalized using a percentile-based rescaling procedure to reduce variation across scans and place images on a comparable numerical scale. Specifically, for each two-dimensional slice, the 1st and 99th percentile intensity values were computed. Voxel intensities were clipped to the [1st percentile, 99th percentile] range and then linearly rescaled to the [0, 1] interval using min–max normalization within the clipped range. This percentile normalization approach was chosen over global z-score normalization because it is more robust to outlier voxels and intensity artifacts that can arise from scanner-specific variations in the OASIS-1 cohort. Each selected slice inherited the subject-level clinical label derived from the corresponding CDR score. Importantly, all partitioning was performed at the subject level rather than at the slice level. This meant that every slice from a given subject remained in the same training, validation, or test subset throughout the analysis, thereby preventing data leakage. Subject-level partitioning was essential because mixing slices from the same subject across subsets would artificially inflate performance by exposing the model to a nearly identical anatomy during both training and evaluation tasks (Figure 1).

#### 2.2.3. Model Architecture and Development

A two-dimensional convolutional neural network (2D CNN) was implemented in TensorFlow/Keras and trained separately for each binary classification task using CDR scores as labels. Four pairwise analyses were defined: CN vs. AD, MCI vs. AD, CN vs. MCI, and CN vs. any impairment (MCI + AD). The network received individual axial MRI slices (256 × 256 × 1 grayscale) as input. Online data augmentation was applied during training, consisting of random horizontal flipping and random rotation (up to ±5%), to increase effective training variation and reduce overfitting. Data augmentation was confirmed to be active in all training runs and is depicted in the model architecture schematic (Supplementary Figure S1).

The architecture consisted of four successive convolutional blocks. Each block contained one convolutional layer with 3 × 3 kernels, “same” padding, and ReLU activation, followed by 2 × 2 max pooling. The four convolutional layers used 16, 32, 64, and 128 filters, allowing the network to learn spatial image features at increasing levels of abstraction. After the final pooling layer, a dropout layer (rate = 0.35) was applied for regularization. The resulting feature maps were then flattened and passed to a fully connected dense layer with 128 units and ReLU activation, followed by an additional dropout layer (rate = 0.35), and a task-specific SoftMax output layer with 2 units that produced class probabilities for the two target groups.

Model optimization was performed with the Adam optimizer (learning rate = 1 × 10^−4^) and sparse categorical cross-entropy loss. Training was conducted for a maximum of 60 epochs with a batch size of 16. Early stopping based on validation loss was used during training with a patience of 10 epochs, and the model weights with the best validation performance were restored. To account for class imbalance in the training data, class-specific sample weights were applied, with the minority class receiving a weight proportional to the inverse class frequency scaled by a factor of 1.5. Within each training fold, 20% of the training subjects were reserved as an internal validation subset for early stopping. A compact 2D CNN was selected intentionally because the primary aim of this study was not to maximize model size, but to test whether a comparatively simple deep learning architecture could recover a meaningful structural signal from a small MRI dataset. We call this model the “from-scratch model” hereafter, to distinguish from the transfer learning model as described below. A schematic diagram of the full model architecture and analysis pipeline is provided in Supplementary Figure S1.

In addition to the from-scratch network model, a transfer learning model was evaluated identically. A MobileNetV2 pretrained on ImageNet was used as a frozen feature extractor in place of the convolutional stack, with slices resized to 224 × 224 and passed to a matched dense head. In the from-scratch model, all convolutional weights are learned from the training folds, whereas in the transfer model, the pretrained features are imported and frozen so that only the small classification head is trained. Identical inputs, augmentation, and evaluation isolate the value of the pretrained representation. A deliberately compact from-scratch architecture was chosen so that measured performance reflects the signal recoverable from the small cohort itself rather than the capacity transferred from large external datasets. The transfer model then quantifies how much additional signal pretrained features add, an approach shown to help small-sample AD-MRI classification [9,12].

#### 2.2.4. Model Evaluation

Model performance was assessed with subject-level 5-fold cross-validation repeated 10 times, with subjects (not slices) partitioned into five mutually exclusive folds so that every slice from a given subject stayed in a single fold. In each iteration, four folds were used for training and the remaining fold served as the held-out test set, and each subject was evaluated once per repeat. Subject-level predictions were formed by soft voting: for a subject i with slices s = 1…S (S = 7), the aggregated positive-class probability was ${\bar{p}}_{i}$ = (1/S) Σ_s_ p_i,s_, and the subject was assigned to the positive class when ${\bar{p}}_{i}\geq \tau$. Averaging probabilities before thresholding (rather than majority-voting hard slice labels) suppresses per-slice noise and yields one decision per subject, which is the clinically relevant unit.

To avoid optimistic bias, the decision threshold τ was selected exclusively on an inner validation split within each training fold and then applied unchanged to the held-out test fold; no test-fold information informed threshold choice. Combined with strict subject-level partitioning, this removes both slice-level leakage and threshold tuning on test data. Discrimination was summarized by the area under the receiver-operating-characteristic curve (ROC-AUC) with 95% confidence intervals (CIs) across the repeats, and each task was tested against the 0.5 chance level with a permutation test. Subject-level accuracy, balanced accuracy (the mean of class-wise recalls), and class-wise recall (sensitivity and specificity) were also reported, and confusion matrices were retained as a descriptive summary. Probability calibration (reliability curves and Brier scores) and decision curve analysis were computed post hoc from the stored subject-level probabilities as supplementary analyses.

#### 2.2.5. Dendrogram Similarity Analysis

To quantify relative similarity among the diagnostic groups, each pairwise binary task was summarized by its model confusion matrix and converted into a symmetric mutual-confusion distance [14]. For a pair of groups (a, b), let the row-normalized off-diagonal error rates be e(a→b) and e(b→a)—the fraction of true-a subjects predicted b, and vice versa, using row-normalized rates rather than raw accuracy controls for the unequal class sizes across tasks. Pairwise similarity was defined as the mean mutual misclassification, s(a,b) = ½[e(a→b) + e(b→a)], and the corresponding dendrogram distance as d(a,b) = BA(a,b), the balanced accuracy of the a-versus-b classifier, so that more-confused pairs sit closer. The three pairwise distances were assembled into a symmetric matrix and clustered by hierarchical agglomerative clustering with average linkage; after groups a and b merge, the distance to a third group c is ½[d(a,c) + d(b,c)]. Smaller values therefore indicate greater similarity (greater mutual confusion) and larger values indicate stronger separability. Critically, this encodes confusion-derived similarity, not a geometric or clinical distance: it describes how often the trained classifiers confused each pair under matched conditions, and any clinical reading is hypothesis-generating only. The one-versus-rest task (CN vs. MCI + AD) was used only as a supplementary check and not included in the clustering, because the three-group tree is fully determined by the three pairwise distances.

#### 2.2.6. External Validation on an Independent AD Cohort (OASIS-2)

To assess model generalizability beyond the primary OASIS-1 cohort, the trained models were additionally evaluated using an independent AD cohort using the OASIS-2 dataset [15]. The OASIS-2 analytic cohort comprised 150 subjects, binned by CDR exactly as in OASIS-1 (85 CN [CDR = 0], 52 MCI [CDR = 0.5], and 13 AD level [CDR ≥ 1]), accordingly. The external binary test sets comprised 98 subjects for CN vs. AD, 65 for MCI vs. AD, 137 for CN vs. MCI, and all 150 for CN vs. any impairment. The OASIS-2 dataset was originally described by Marcus et al. [15]. As OASIS-2 is a longitudinal dataset, a single MRI scan was selected for each participant so that each individual contributed only one subject-level observation, ensuring independence among test samples. The OASIS-2 MRIs were pre-processed using a similar procedure as described for OASIS-1, including procedures for intensity normalization, slice-selection, resizing, and subject-level aggregation, ensuring that performance differences reflected dataset transfer rather than a change in pre-processing. No model weights, thresholds, or hyperparameters were updated when using OASIS-2. Instead, the OASIS-1-trained models were applied to OASIS-2 without retraining, thereby treating OASIS-2 as a fully external test cohort. External discrimination was summarized at the subject level by ROC-AUC with 95% CI and permutation tests against the 0.5 chance level for each binary task.

#### 2.2.7. Calibration and Decision Curve Analysis

Beyond discrimination, probabilistic calibration was examined post hoc from the stored subject-level predicted probabilities. For each binary task, reliability curves were generated by comparing mean predicted risk with observed event frequency across probability bins, and the Brier score was calculated as BS = (1/N) Σ_i_ (p_i_ − y_i_)^2^, where p_i_ is the subject-level predicted probability and y_i_ is the observed binary outcome. Because class-weighted training can distort raw probability estimates, these calibration analyses were interpreted as supplementary rather than primary model-evaluation measures. To further evaluate the model’s potential clinical utility, decision curve analysis (DCA) was performed across a range of threshold probabilities. Net benefit was calculated as NB(*t*) = TP/N − FP/N × [*t*/(1 − *t*)], where *t* is the threshold probability. The resulting decision curves were compared with the default “treat-all” and “treat-none” strategies to assess whether the model offered a clinically meaningful advantage over conventional decision rules.

#### 2.2.8. Class-Imbalance and Mitigation Ablation Studies

Because the AD group was substantially smaller than the CN and MCI groups, an ablation analysis was conducted to distinguish the effect of class imbalance from those associated with a limited sample size. This comparison was restricted to the AD-inclusion tasks (CN vs. AD and MCI vs. AD). Three class-mitigation strategies were evaluated using the same leak-free subject-level repeated cross-validation framework: (i) inverse-frequency class weighting, which served as the baseline imbalance-mitigation strategy; (ii) minority oversampling applied within the training folds only; and (iii) transfer learning with a pretrained two-dimensional encoder and a trainable classification head. For the class-weighted conditions (the inverse-frequency baseline and the transfer arm), the minority AD class was assigned an additional 4.5-fold weighting beyond that determined by inverse-frequency balancing to further compensate its underrepresentation. For the oversampling condition, we applied resampling with no class weights. The validation and test sets were not resampled, ensuring that only the training distribution differed across the evaluated strategies. All other components of the pipeline, including subject-level partitioning, pre-processing, slice aggregation, and evaluation metrics, were kept constant. Performance was summarized by ROC-AUC, balanced accuracy, and AD positive-class recall.

#### 2.2.9. Augmentation and Self-Supervised Pretraining Evaluation

To evaluate whether a more data-efficient training method could improve the from-scratch model under the small-data setting, we next evaluated augmentation strategies and performed additional ablation studies on the CN vs. AD classification task using the same leak-free evaluation framework. Three training strategies were compared. (i) The baseline augmentation strategy, used in the primary from-scratch model, consisted of random horizontal flipping and small-angle rotations. (ii) A mixed-augmentation strategy incorporated random translations, zooming, contrast perturbations, and Gaussian noise. (iii) A self-supervised learning strategy first pretrained the encoder using a rotation-prediction task on training-fold slices, followed by supervised fine-tuning for the downstream CN versus AD classification task. Subject-level data splits, optimization parameters, and evaluation metrics were held constant across all experiments, allowing the independent effects of data augmentation and self-supervised pretraining to be evaluated. Augmentation was applied online during training using the following magnitudes: (i) the baseline strategy used random horizontal flipping (50% probability) and random rotation of up to +/−0.05 of a full turn (approximately +/−18 degrees); (ii) the mixed strategy additionally used random rotation up to +/−0.08 (approximately +/−29 degrees), random translation up to +/−8% of the image height and width, random zoom of +/−10%, random contrast adjustment (factor 0.2), and additive Gaussian noise (standard deviation of 0.03 on the [0,1]-normalized intensities); and (iii) the self-supervised pretext task classified each slice into one of four discrete rotations (0, 90, 180, and 270 degrees) before the encoder was fine-tuned on the downstream task.

#### 2.2.10. Three-Dimensional CNN Model Evaluation

The conversion of three-dimensional MRI volumes into two-dimensional slices may discard spatial context and anatomical information. To evaluate if this impacts model performance, we trained a compact three-dimensional convolutional neural network (3D CNN) on the whole down-sampled T1-weighted volumes for the CN vs. AD classification task and compared this with the 2D CNN under the same evaluation framework. The MRI volumetric inputs were preprocessed at the subject level and evaluated using the same subject-level folds used for the two-dimensional models. The 3D architecture was intentionally kept compact to remain consistent with this study’s small-data framing and to ameliorate overfitting. Model performance was summarized by subject-level ROC-AUC and compared descriptively with the two-dimensional from-scratch and transfer learning models. Prior to modeling, each volume was resampled to 96 × 96 × 64 voxels by linear interpolation and intensity-normalized to its 1st-99th percentile. The 3D CNN comprised three volumetric convolutional blocks with 16, 32, and 64 filters (3 × 3 × 3 kernels, ReLU activation, same padding), each followed by 2 × 2 × 2 max pooling; a global average pooling layer; and a classification head consisting of a 64-unit ReLU dense layer and a two-way SoftMax output, with dropout (rate = 0.4) applied before and after the dense layer. The network was trained with the Adam optimizer (learning rate of 1 × 10^−4^) and sparse categorical cross-entropy loss for up to 40 epochs (batch size of 8) with early stopping (patience: 8, best weights restored) and the same inverse-frequency class weighting with an additional 4.5-fold minority up-weighting used for the 2D models.

#### 2.2.11. Visualization of Model Attention via Grad-CAM

To identify the image regions contributing most strongly to model predictions, Gradient-weighted Class Activation Mapping (Grad-CAM) [16] was applied to the transfer learning 2D CN versus AD model. For this qualitative interpretability analysis, the transfer learning model was trained using a single subject-level 80/20 train–test split rather than the full repeated cross-validation framework. This approach facilitated the generation of Grad-CAM visualizations for individual subjects, with representative examples selected preferentially from the held-out test set.

Grad-CAM saliency maps were generated for representative, correctly classified CN and AD subjects by computing gradients with respect to the predicted class. The resulting activation maps were normalized and overlaid on the corresponding central axial T1-weighted MRI slice to identify the brain regions driving the model’s decisions. In addition to representative individual examples, mean Grad-CAM saliency maps were computed across correctly classified AD subjects (22 of the 25 AD subjects) to visualize recurring attention patterns. These saliency maps were used only as a qualitative interpretability analysis and were not interpreted as direct evidence of localized atrophy or disease-specific pathology.

## 3. Results

### 3.1. Characteristics of the Analyzed OASIS-1 Cohort

The pipeline for current analysis is given in Figure 1. After the exclusion of subjects without valid Clinical Dementia Rating (CDR) labels, the final analytic cohort consisted of 214 subjects, each contributing one structural MRI volume, seven central axial slices (1498 slices total), and one subject-level clinical label. The filtered dataset contained 124 cognitively normal (CN) subjects; 65 subjects with CDR = 0.5, operationalized here as mild cognitive impairment (MCI); and 25 subjects with CDR ≥ 1, grouped as the AD category for analysis. This class distribution is important for interpreting the model’s results because the dataset was not class-balanced: CN accounted for 57.9% of the cohort, MCI for 30.4%, and AD for only 11.7%. Accordingly, the binary tasks were built on different sized subsets, including 149 subjects for CN vs. AD, 90 for MCI vs. AD, 189 for CN vs. MCI, and all 214 subjects for CN vs. MCI + AD.

This sampling structure has direct implications for performance interpretation. Tasks involving AD were affected by the smallest class size, making disease-class sensitivity more vulnerable to modest changes in prediction counts. At the same time, the repeated subject-level 5-fold cross-validation design still allowed the model to be tested under a consistent evaluation framework across tasks. The resulting findings should therefore be interpreted as a small-data feasibility assessment of what a lightweight structural MRI CNN can recover from a modest public cohort, rather than as a definitive estimate of clinically deployable performance.

### 3.2. Overall Subject-Level Classification Performance Across Tasks

**Figure 2:**
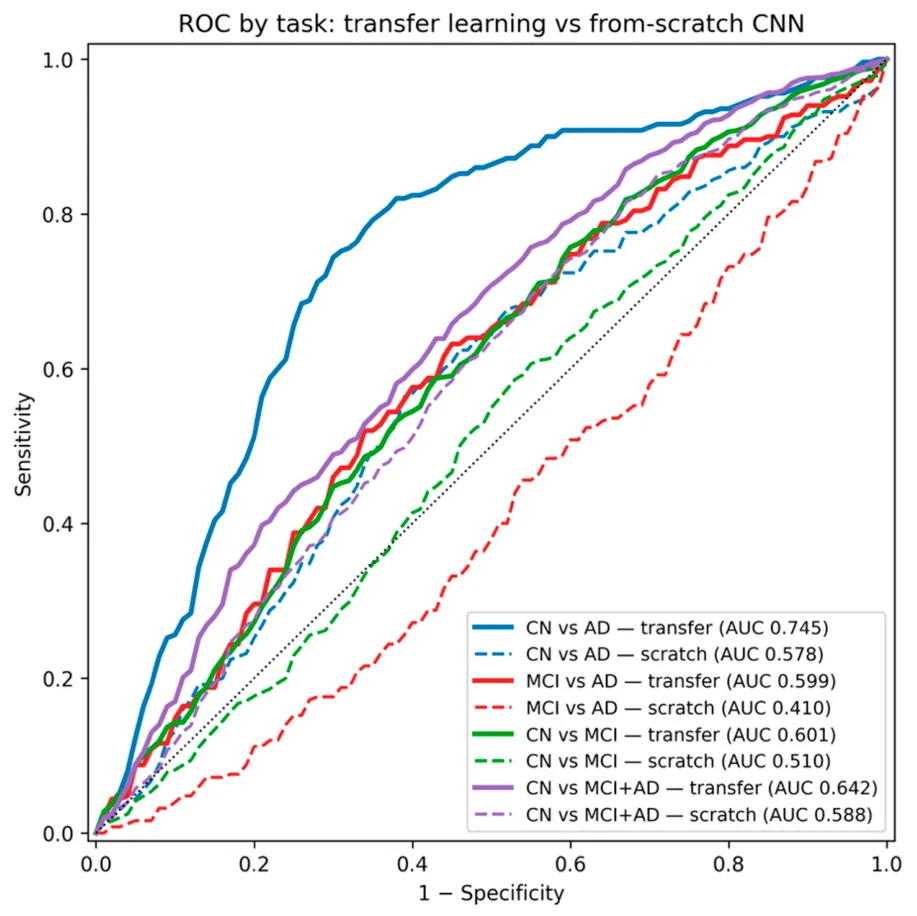
Subject-level ROC curves for all four binary tasks using transfer learning (solid) versus from-scratch (dashed) models. Each curve represents the mean ROC across the 10 cross-validation repeats, and the AUC values are the mean-over-repeats values reported in Table 1. Transfer learning raises every curve, most clearly for CN vs. AD.

Under the leakage-controlled repeated protocol, discrimination is summarized by ROC-AUC for the from-scratch and transfer models across the four tasks (Table 1, Figure 2). Both models share the same broad ordering—normal versus impaired and CN vs. AD are easier than the adjacent-stage tasks—but transfer learning shifts the whole ROC upward. The from-scratch CNN recovered only a coarse normal-versus-impaired signal (CN vs. any impairment AUC 0.59, 95% CI 0.53–0.63; *p* = 0.004) and was at chance on the adjacent-stage contrasts (MCI vs. AD 0.41, *p* = 0.98; CN vs. MCI 0.51, *p* = 0.38). The transfer model exceeded chance on every task, most clearly for CN vs. AD (AUC 0.745, 95% CI 0.730–0.763; *p* = 0.0002). The earlier impression that MCI vs. AD was the most separable task did not survive the removal of test-fold threshold tuning: under threshold-independent ROC-AUC, it is in fact the weakest transfer contrast (0.599). This internal ordering should be read together with the external validation in Section 3.9.

### 3.3. CN Versus AD Classification Performance

For CN vs. AD, ROC-AUC was 0.58 (95% CI 0.54–0.62; *p* = 0.069) from scratch and 0.745 (95% CI 0.730–0.763; *p* = 0.0002) for transfer—the strongest and most stable result in this study. Even so, the aggregated confusion matrices (Supplementary Figure S2) show that both models still misclassified a substantial minority of CN as AD and missed roughly half of the AD subjects, so even the best-separated pair carried two-way errors and low AD precision. The transfer model’s clearer separation is visible as the uppermost ROC curve in Figure 2.

### 3.4. MCI Versus AD Classification Performance

For MCI vs. AD, the from-scratch model was at chance (AUC 0.41; *p* = 0.98) and transfer reached only 0.599 (*p* = 0.031). This contrast appeared strongest under the earlier accuracy-with-threshold-tuning analysis, but that impression reflected threshold selection on the scored subjects; under threshold-independent ROC-AUC, it is the weakest transfer task. Persistent MCI AD confusion (Supplementary Figure S2) is consistent with the similarity analysis below, in which these two impaired stages are the least separable pair.

### 3.5. CN Versus MCI Classification Performance

CN vs. MCI showed the greatest contrast: AUC of 0.51 (*p* = 0.38) from scratch and 0.601 (*p* = 0.004) for transfer learning. Even with pretrained features, the model only modestly exceeded chance, indicating that structural MRI in this small-data setting does not carry sufficiently distinctive information to resolve the earliest impairment boundary; the heavy overlap between CN and MCI dominates the error structure (Supplementary Figure S2).

**Figure 3:**
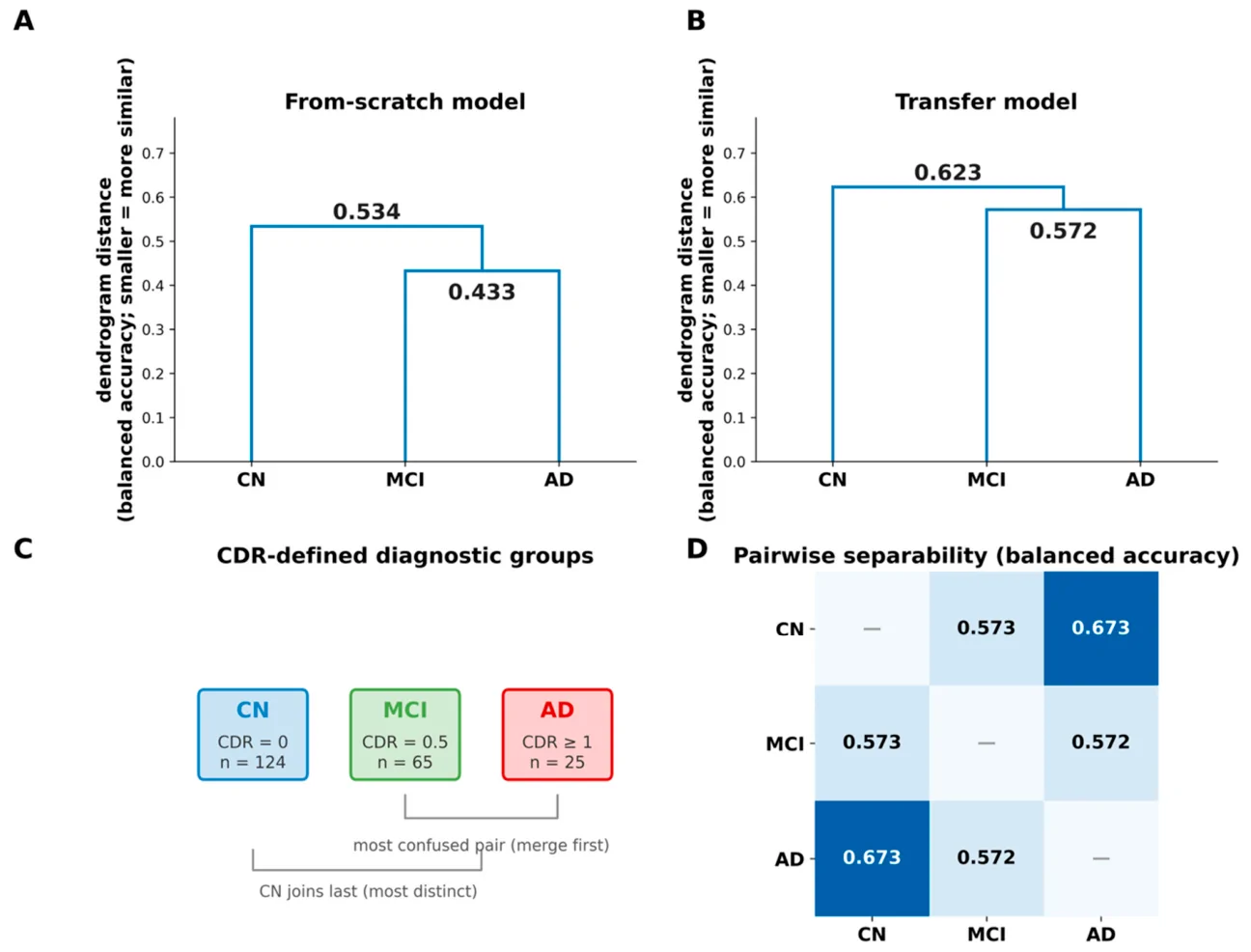
Classifier-derived similarity analysis among CN, MCI, and AD groups. Dendrograms were generated using pairwise balanced accuracy as the distance metrics, where lower values indicate greater mutual confusion between groups and therefore higher similarity. (**A**) Dendrogram for the from-scratch model showing a coarse separation in which CN appears as an outlier relative to MCI and AD. This should be interpreted with caution since the pairwise classifiers perform below chance (for example, MCI vs. AD balanced accuracy of 0.433). (**B**) Dendrogram for the transfer learning model, in which MCI and AD merge first (the most mutually confused pair) and CN joins last as the outlier, giving the topology ((MCI, AD), CN), with pairwise distances of 0.673 (CN vs. AD), 0.573 (CN vs. MCI), and 0.572 (MCI vs. AD). (**C**) The three CDR-defined diagnostic groups (CN, CDR = 0, *n* = 124; MCI, CDR = 0.5, *n* = 65; AD, CDR *≥* 1, *n* = 25), annotated with the transfer-model merge order: MCI and AD form the most confused pair and CN is the most distinct group. (**D**) Pairwise separability matrix showing the transfer-model balanced accuracies: CN vs. MCI 0.573, CN vs. AD 0.673, and MCI vs. AD 0.572, with the diagonal undefined. Dendrograms represent the classifier-derived measures of separability obtained under identical training and evaluation conditions. These measures should be interpreted as the model’s ability to distinguish between groups rather than geometric distances in feature space or clinical distances between disease states.

### 3.6. CN Versus Any Impairment (MCI + AD) Classification Performance

For CN versus any impairment (MCI + AD), ROC-AUC was 0.59 (95% CI 0.53–0.63; *p* = 0.004) from scratch and 0.642 (*p* = 0.0002) for transfer. Pooling the impaired groups produced a more symmetric, broadly abnormal-versus-normal contrast that both models detected above chance, though still too weak for stand-alone screening. Its supplementary one-versus-rest separability (d = 0.210) is reported for completeness but is not an edge in the three-group dendrogram.

### 3.7. Confusion-Derived Similarity Structure and Hierarchical Clustering

To compare CN, MCI, and AD on a common classifier-derived scale, each pairwise confusion matrix was converted into a row-normalized mutual-confusion distance (Section 2.2.5), where the smaller distances indicate greater similarity. The resulting balanced-accuracy separability matrix is given in Figure 3D.

Hierarchical agglomerative clustering (average linkage) was applied to both models, and the two-panel dendrogram is shown in Figure 3. In the transfer model, the pairwise dendrogram distances (balanced accuracy) were 0.673 (CN vs. AD), 0.573 (CN vs. MCI), and 0.572 (MCI vs. AD): CN vs. AD is the most separable pair, so CN forms the outlier and joins last, while MCI and AD are the most mutually confused (near-tied with CN vs. MCI) and merge first, giving the topology ((MCI, AD), CN). The from-scratch model produced the same CN-as-outlier shape but is unreliable for the fine structure, being at or below chance on the pairwise tasks (for example MCI vs. AD balanced accuracy 0.43). Because these distances are confusion-derived, smaller values indicate greater similarity (more mutual misclassification), not a geometric or clinical distance, and any reading of the topology is a property of the model under matched conditions rather than a measurement of biological similarity.

### 3.8. Cross-Task Misclassification Patterns and AD Stage-Dependent Separability

Taken together, the confusion matrices and the dendrogram reveal a coherent stage-dependent pattern. Discrimination was strongest for CN vs. AD, intermediate for CN vs. any impairment, and weakest for the two adjacent-stage boundaries, CN vs. MCI and MCI vs. AD, on which the transfer model only modestly exceeded chance and the from-scratch model was at chance. The clustering quantified the same trend on a unified scale: in the transfer model, MCI and AD formed the earliest-merging (most mutually confused) pair while CN was the most distinct group and joined last, with MCI sitting almost equidistant between CN and AD. The errors were not random—the model repeatedly confused neighboring stages (CN with MCI, MCI with AD)—implying that the learned MRI features reflect a continuum rather than sharply discrete categories. For a small public dataset and a lightweight pipeline, this is a meaningful result: the models extracted a nontrivial CDR-defined cognitive-status signal from structural MRI, but that signal was strongest for the widest contrast (CN vs. AD) and weakest for the earliest, most clinically ambiguous transitions.

### 3.9. External Validation on an Independent Cohort (OASIS-2)

To further validate our findings, we expanded our analysis to the OASIS-2 dataset [15]. OASIS-2 is a publicly available longitudinal MRI dataset comprising cognitively normal older adults and individuals with mild to moderate Alzheimer’s disease, with multiple imaging sessions acquired over time using a different scanner and protocol generation. The OASIS-1-trained models were applied without retraining to this independent cohort using one scan per subject. The transfer CN vs. AD model reached AUC = 0.748 (95% CI = 0.628–0.872; permutation *p* = 0.0006), essentially identical to its internal estimate (0.745), and all eight model/task combinations exceeded chance (Table 2). The internal ordering did not fully replicate externally: transfer retained a clear edge only for CN vs. AD, while the from-scratch model was comparable or better on the adjacent-stage tasks. The external CIs are wide (only 13 AD subjects in OASIS-2), so task-level differences should be read cautiously; the key result is that the headline CN vs. AD performance reproduced on a fully independent cohort.

### 3.10. Model Evaluation and Decision Curve Analysis

In addition to evaluating discriminative performance, we assessed probability calibration, which measures how well the model’s predicted probabilities correspond to the true likelihood of disease. Calibration was evaluated using reliability diagrams, which compare predicted probabilities with observed outcome frequencies, and Brier scores, which quantify the overall accuracy of probabilistic predictions, with lower scores indicating better calibration. Both metrics were computed from the subject-level predicted probabilities.

Transfer learning Brier scores were 0.230 (CN vs. AD), 0.313 (MCI vs. AD), 0.379 (CN vs. MCI), and 0.335 (CN vs. any impairment). Since the Brier score reflects both calibration and class prevalence, it should be interpreted together with AUCs rather than in isolation.

**Figure 4:**
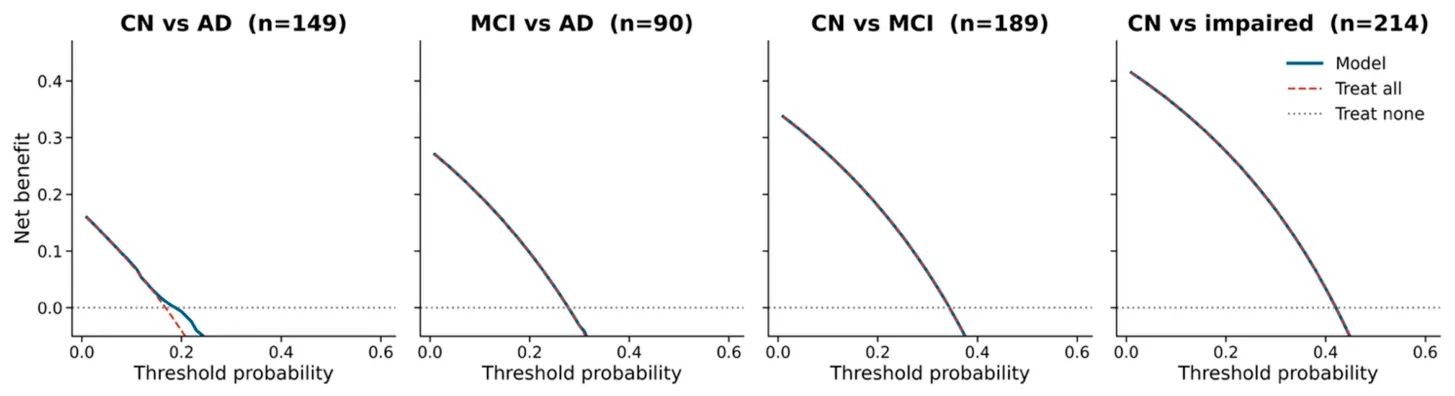
Decision curve analysis for the transfer learning model across the four classification tasks. Net benefit is plotted against threshold probability, with treat-all (dashed) and treat-none (dotted) strategies shown for comparison. Clinical utility is observed when the model curve exceeds both reference strategies. A modest net benefit was observed for the CN versus AD task over threshold probabilities in the range of approximately 0.15–0.25, whereas the remaining tasks showed little or no advantage over the treat-all strategy.

To assess clinical utility, decision curve analysis (DCA) was performed to determine whether the model provided a greater net benefit than the default “treat-all” and “treat-none” strategies across clinically relevant threshold probabilities. Figure 4 shows DCA analysis, showing the net benefit as a function of threshold probability for “treat-all “and “treat-none” reference strategies. A model adds value only when its decision curve lies above both reference strategies [17]. Across the four tasks (Figure 4), the transfer model showed a small positive net benefit over both baselines only for CN vs. AD, and only within a narrow threshold band of roughly 0.15–0.25. For MCI vs. AD, CN vs. MCI, and CN vs. any impairment, the curves essentially coincided with the treat-all strategy. DCA therefore supports a cautionary reading: any potential clinical utility is limited to the easiest contrast (CN vs. AD) and is modest, consistent with the AUC values, and this model is not suitable for stand-alone clinical use.

### 3.11. Class-Imbalance Mitigation

To assess the impact of class imbalance in this small dataset, we compared two imbalance-mitigation strategies, inverse-frequency class weighting, and minority oversampling using the models trained from scratch. We additionally evaluated a transfer learning model using the same repeated subject-level cross-validation framework to determine whether pretrained representations provided further improvements. Performance was assessed using ROC-AUC, balanced accuracy, and AD (positive-class) recall. For the CN versus AD task, the inverse-frequency class-weighting baseline performed near chance (AUC = 0.506, AD recall = 0.20). Minority oversampling improved discrimination (AUC = 0.606, AD recall = 0.40), whereas transfer learning yielded the best overall performance (AUC = 0.728, balanced accuracy = 0.657, and AD recall 0.52, consistent with the main transfer result of 0.745). The same ordering held on MCI vs. AD, though every strategy remained weak (AUC = 0.514, 0.468, and 0.547; AD recall rising from 0.01 under class weighting to 0.47 under transfer). These results indicate that although class-imbalance mitigation influences model performance, its effect is modest compared with that of transfer learning. While minority oversampling improved CN versus AD discrimination relative to inverse-frequency class weighting alone, only transfer learning produced a substantial performance gain. This finding suggests that, in the current small-data setting, the primary limitation is the model’s ability to learn robust feature representations rather than class imbalance alone. Our results are consistent with recent Alzheimer’s disease MRI studies demonstrating that pretrained representations, together with appropriate imbalance-mitigation strategies, improve balanced accuracy and overall classification performance [11].

### 3.12. Augmentation and Self-Supervised Pretraining Studies

We also evaluated the impact of data augmentation strategies on model performance. The baseline augmentation strategy used in the primary from-scratch model (random horizontal flipping and small-angle rotations) was compared with the mixed-data augmentation strategy (random translations, zooming, contrast perturbations, and Gaussian noise) and a self-supervised learning strategy in which the encoder was first pretrained using a rotation-prediction task on training-fold slices before supervised fine-tuning for the downstream CN versus AD classification task. All strategies were evaluated under the same leak-free, subject-level framework to isolate the effects of data augmentation and self-supervised pretraining.

Overall gains were marginal: relative to the baseline (AUC = 0.505), mixed augmentation reached 0.517 (+0.012) and self-supervised pretraining 0.521 (+0.016), with balanced accuracy essentially unchanged near 0.50. Data-efficient augmentation and self-supervised pretraining therefore provided only small improvements and did not overcome the small-data ceiling for a from-scratch encoder. These results are consistent with the imbalance ablation studies performed in which the substantial gains came from transfer learning rather than from training-time strategies [10].

### 3.13. Three-Dimensional CNN Evaluation

To evaluate whether converting three-dimensional MRI volumes into two-dimensional slices resulted in a loss of diagnostically relevant information, we trained a compact 3D CNN on down-sampled whole-brain T1-weighted volumes using the same subject-level data partitions. This enabled a direct comparison of the 3D and 2D models under an identical evaluation framework. On CN vs. AD, it reached an AUC of 0.525 (permutation *p* = 0.39), which is not above chance and does not exceed either the 2D from-scratch model (0.58) or the 2D transfer learning model (0.745). With the current small sample size, the increased representational capacity of a volumetric 3D CNN was offset by a greater tendency to overfit, rather than recovering information potentially lost during slice-based processing. These findings support the use of a lightweight 2D CNN as an appropriate baseline for small-data structural MRI studies and suggest that volumetric 3D models are more likely to be beneficial in larger cohorts. This conclusion is consistent with recent studies demonstrating that 2D slice-based representations can preserve most of the diagnostically relevant information contained in full MRI volumes while substantially reducing computational complexity [10].

### 3.14. Model Attention via Grad-CAM

To identify the brain regions that most strongly influenced model predictions, Gradient-weighted Class Activation Mapping (Grad-CAM) [16] was applied to the transfer learning CN versus AD model on a representative set of correctly classified test subjects (Supplementary Figure S3). In correctly classified AD subjects, model attention was concentrated in the central periventricular brain regions and distributed broadly across the cerebrum rather than being confined to a single anatomic structure. Additionally, Grad-CAM saliency maps averaged across all correctly classified AD subjects showed that model attention was consistently concentrated in central periventricular regions. This pattern is broadly consistent with the ventricular enlargement and generalized cerebral atrophy commonly observed in Alzheimer’s disease, although the saliency maps should be interpreted as reflecting model attention rather than direct evidence of pathology. In correctly classified cognitively normal subjects, attention over the brain interior was comparatively weak.

Some attention nonetheless was located near the brain boundaries and extra-cerebral regions in some of the individual cases, indicating that model attention likely keys on global brain-shape and cerebrospinal-fluid-space cues (ventricular and sulcal widening) rather than a specific medial-temporal focus. These maps therefore indicate model focus only and should not be read as direct evidence of localized brain atrophy. These findings are consistent with the coarse, stage-dependent structural signal observed in this study, whereby advanced disease is more readily distinguished by gross atrophic changes than the subtle anatomical alterations present at the CN–MCI boundary.

## 4. Discussion

This study investigated how many Alzheimer’s disease-related signals can be recovered from a small structural MRI cohort using a lightweight 2D convolutional neural network (2D CNN) and whether transfer learning improves classification performance.

Our from-scratch 2D CNN recovered only a coarse distinction between cognitively normal and cognitively impaired subjects and performed at chance when distinguishing adjacent disease stages. In contrast, the transfer learning model, initialized with frozen ImageNet features, consistently improved performance across all classification tasks and achieved an AUC of 0.745 for CN versus AD on the internal dataset and 0.748 on the independent OASIS-2 cohort.

Modeling strategy was a key determinant of performance, as pretrained representations extracted considerably more Alzheimer’s disease-related signals than a from-scratch network when trained on a small dataset.

A rigorous evaluation was equally important. Although threshold tuning suggested that the from-scratch model performed best for MCI versus AD classification, threshold-independent evaluation using AUC revealed this to be the weakest classification task. Moreover, the overall performance remained modest, with discrimination between CN and MCI approaching chance levels. Collectively, these findings highlight the limitations of structural MRI classification in small datasets and indicate that the results should be viewed as a methodological feasibility study rather than evidence of clinical utility or reliable detection of Alzheimer’s disease pathology.

In practical terms, this study suggests that small-data MRI deep learning may be more useful for coarse severity discrimination than for early stand-alone diagnosis. The weak CN vs. MCI result showed that structural MRI alone, at least in this limited-sample context, does not provide sufficiently distinctive information to resolve subtle early impairment reliably. By contrast, the somewhat stronger MCI vs. AD and CN vs. MCI + AD results indicate that the model could detect a broader abnormality signal once neurodegenerative burden became more established or when impaired groups were pooled.

The dendrogram similarity analysis adds a useful interpretive layer. A confusion matrix shows where errors occur in a single task; the similarity analysis converts those task-specific errors into a unified geometry across CN, MCI, and AD. In the transfer model, MCI and AD formed the most mutually confused pair while CN was the most distinct group, so in the learned classifier space the two impaired stages were the hardest to separate and MCI sat almost equidistant between normal aging and Alzheimer-level impairment. This neither proves a biological progression pathway nor implies that the learned structure is pathology-specific; these distances describe how often the classifiers confused each pair under matched conditions, not clinical or biological similarity. It is nonetheless informative because it quantifies why MRI-only deep learning remains limited for early case finding, even when a disease-related signal is present.

This stage-dependent pattern is also consistent with what has been reported in larger MRI deep learning studies, where broader cognitively normal-versus-impaired contrasts tend to outperform the harder task of identifying mild impairment. The similarity of the qualitative pattern across small and large datasets suggests that the challenge observed here is not merely a technical artifact of a weak model. Instead, it likely reflects a genuine property of structural MRI in Alzheimer-spectrum classification: later disease carries a stronger and more spatially consolidated neurodegenerative signal, whereas the earliest changes overlap heavily with normal aging and other sources of clinical heterogeneity.

This study has several strengths despite its modest scale. It uses an openly available public dataset, a transparent and computationally accessible 2D CNN pipeline, and subject-level evaluation intended to reduce leakage. These features make the work reproducible and relevant for groups that do not have access to very large imaging consortia or extensive computing infrastructure. The new similarity analysis also improves the interpretability of the results by summarizing the relative closeness of the diagnostic groups rather than reporting only task-specific accuracies. For a small-data study, this kind of modest model is clinically valuable in its own right.

Several limitations temper the interpretation of our results. The most important is sample size and class imbalance: the OASIS-1 cohort included only 25 AD subjects, making the AD category the smallest and least stable to estimate and contributing to low AD precision and the persistent misclassification of AD as MCI. Because CDR labeling was administered only to older adults, the analytic cohort is also older and narrower in age than the full OASIS-1 release; this is a deliberate label-availability constraint rather than a random sample and may bias the cohort toward an older, screened population, limiting generalization to younger or unscreened groups. Although external validation on OASIS-2 (Section 3.9) partly addresses the single-cohort concern, both cohorts share the OASIS acquisition lineage, so validation on an independent consortium (for example ADNI or AIBL) remains desirable. A further limitation concerns label definition: CDR = 0.5 is a clinically heterogeneous category that need not reflect prodromal Alzheimer’s disease, and without amyloid or tau confirmation the MCI group may include vascular, psychiatric, or other non-AD contributors, so weak CN–MCI separability is partly attributable to genuine label heterogeneity rather than model failure alone. Accordingly, the model should be interpreted as classifying CDR-defined cognitive-status patterns visible on MRI, not as detecting Alzheimer pathology directly.

The dendrogram similarity analysis also has limitations that should be stated explicitly. The tree was derived from pairwise confusion matrices produced by separate binary tasks. Although the same architecture and evaluation procedure were used throughout, the task-specific models were still trained on different sample subsets with different class balances. For that reason, the dendrogram is best viewed as a standardized summary of classifier-based separability under matched modeling conditions, not as an intrinsic anatomical distance map. The one-vs-rest CN vs. MCI + AD result should likewise be interpreted separately as a normal-versus-disease summary rather than as a fourth edge in the hierarchical tree.

Future work could improve the present study in several directions. The next step toward addressing the small-data question would be assessing whether data-efficient strategies such as transfer learning, self-supervised pretraining, targeted augmentation, and class-imbalance mitigation could help determine whether the current limitations are primarily due to insufficient sample size or to a deeper ceiling of MRI-only separability. Architectural extensions, including 3D CNNs and region-focused analyses of structures such as the hippocampus and entorhinal cortex, may also recover a signal that is lost in a compact slice-based approach. In addition, future studies should prioritize multimodal and longitudinal designs. Combining structural MRI with cognitive testing, demographics, amyloid or tau biomarkers, or longitudinal follow-up would likely improve the interpretability of early-stage predictions and clarify whether borderline cases misclassified by the model are biologically intermediate or simply noisy.

In our view, the contribution of this work is not that it “solves” MRI-based AD classification, but that it defines a boundary of utility for small-data deep learning. The model learned enough from a modest public cohort to separate broader or later-stage impairment better than chance, yet it remained weak at the earliest and most clinically ambiguous transition. The dendrogram similarity analysis sharpened this conclusion by showing that CN was the most distinct group while MCI and AD were the hardest to separate, with MCI almost equidistant from normal aging and Alzheimer-level impairment. That pattern helps explain where the model succeeds, where it fails, and why structural MRI alone remains insufficient for confident early diagnosis in limited-data settings.

## 5. Conclusions

In a small, public, structural-MRI cohort, a from-scratch 2D CNN recovered only a coarse normal-versus-impaired signal and was at chance on finer distinctions, whereas a transfer learning model recovered significantly more signal across all four tasks, reaching CN vs. AD AUC 0.745 internally and 0.748 on independent OASIS-2 under leakage-controlled repeated evaluation. The confusion-derived similarity analysis summarized the learned structure as the topology ((MCI, AD), CN): CN was the most distinct group while the two impaired stages were the least separable, reinforcing that the earliest transition from normal cognition to mild impairment was the hardest boundary.

The main clinical message is cautious. In the small-data regime, data-efficient strategies such as transfer learning are the effective lever, but the resulting model is not a stand-alone diagnostic: labels are CDR-based rather than biomarker-confirmed, so the model separates CDR-defined cognitive-status groups (CN, MCI = CDR 0.5, and CDR ≥ 1 impairment), not Alzheimer pathology per se. Its value is as a transparent, reproducible benchmark used to study where a structural-MRI signal is and is not separable under data constraints. Future progress will likely depend on larger and more diverse cohorts, biomarker-aware and longitudinal labels, richer multimodal inputs, and data-efficient modeling strategies that preserve a more anatomical context.

## Figures and Tables

**Table 1:** Model evaluation. Internal discrimination (ROC-AUC and 95% CI) under leakage-free repeated 5-fold cross-validation (*n* = 10 repeats). CN vs. impaired = CN vs. MCI + AD.

Model	Task	AUC (Mean ± SD)	95% CI	p vs. Chance
From scratch	CN vs. AD	0.58 ± 0.03	0.54–0.62	0.069
Transfer	CN vs. AD	0.745 ± 0.013	0.730–0.763	0.0002
From scratch	MCI vs. AD	0.41 ± 0.06	0.32–0.48	0.98
Transfer	MCI vs. AD	0.599 ± 0.023	0.565–0.633	0.031
From scratch	CN vs. MCI	0.51 ± 0.04	0.43–0.56	0.38
Transfer	CN vs. MCI	0.601 ± 0.024	0.566–0.642	0.004
From scratch	CN vs. impaired	0.59 ± 0.03	0.53–0.63	0.004
Transfer	CN vs. impaired	0.642 ± 0.025	0.604–0.680	0.0002

**Table 2:** Model validation on an independent dataset—the OASIS-2 cohort (no retraining; one scan per subject). CN vs. impaired = CN vs. MCI + AD.

Model	Task	*n*	AUC (Mean ± SD)	95% CI	Perm p
Transfer	CN vs. AD	98	0.748 ± 0.016	0.628–0.872	0.0006
Transfer	MCI vs. AD	65	0.676 ± 0.031	0.492–0.892	0.0144
Transfer	CN vs. MCI	137	0.600 ± 0.013	0.511–0.702	0.0188
Transfer	CN vs. impaired	150	0.607 ± 0.011	0.514–0.692	0.0126
From scratch	CN vs. AD	98	0.681 ± 0.030	0.487–0.865	0.0190
From scratch	MCI vs. AD	65	0.721 ± 0.056	0.579–0.892	0.0024
From scratch	CN vs. MCI	137	0.654 ± 0.093	0.622–0.803	0.0002
From scratch	CN vs. impaired	150	0.663 ± 0.108	0.636–0.801	0.0002

## Data Availability

The data presented in this study are openly available in the Open Access Series of Imaging Studies (OASIS) repository at https://www.oasis-brains.org/, OASIS-1 (cross-sectional) and OASIS-2 (longitudinal) datasets, reference numbers [13,15].

## Supplementary Materials

The following supporting information can be downloaded at: https://www.mdpi.com/article/10.3390/jimaging12080352/s1, Figure S1: Schematic of the 2D CNN architecture used for all binary classification tasks; Figure S2: Subject-level confusion matrices for the from-scratch and transfer models across the four binary tasks; Figure S3: Grad-CAM saliency maps for the transfer learning CN vs. AD model; Table S1: Software packages and versions used in the analysis.

## Abbreviations

The following abbreviations are used in this manuscript:

AD	Alzheimer’s disease
MRI	Magnetic resonance imaging
2D CNN	Two-dimensional convolutional neural network
3D CNN	Three-dimensional convolutional neural network
MCI	Mild cognitive impairment
CN	Cognitively normal
