# Supplementary material for: Small-Data Deep Learning for Alzheimer-Spectrum Classification from Structural MRI: A Feasibility Study Using OASIS
Source: J Imaging. 2026 Aug 3;12(8):352. doi: 10.3390/jimaging12080352 (PMC13514690; doi:10.3390/jimaging12080352)
Supplement: Supplementary file 1 [file jimaging-12-00352-s001.zip › jimaging-4391467.pdf]

Journal of
Imaging

MDPI

Supplementary Materials

Small-Data Deep Learning for Alzheimer-Spectrum Classification from Structural MRI: A Feasibility Study Using OASIS

Ian D. Li, Choong-Yong Ung and Cristina Correia *

Department of Molecular Pharmacology and Experimental Therapeutics, Mayo Clinic College of Medicine and Science, Rochester, MN 55905, USA; ianbubu2008@gmail.com (I.D.L.); ung.choongyong@mayo.edu (C.-Y.U.)
* Correspondence: Correspondence: correia.cristina@mayo.edu

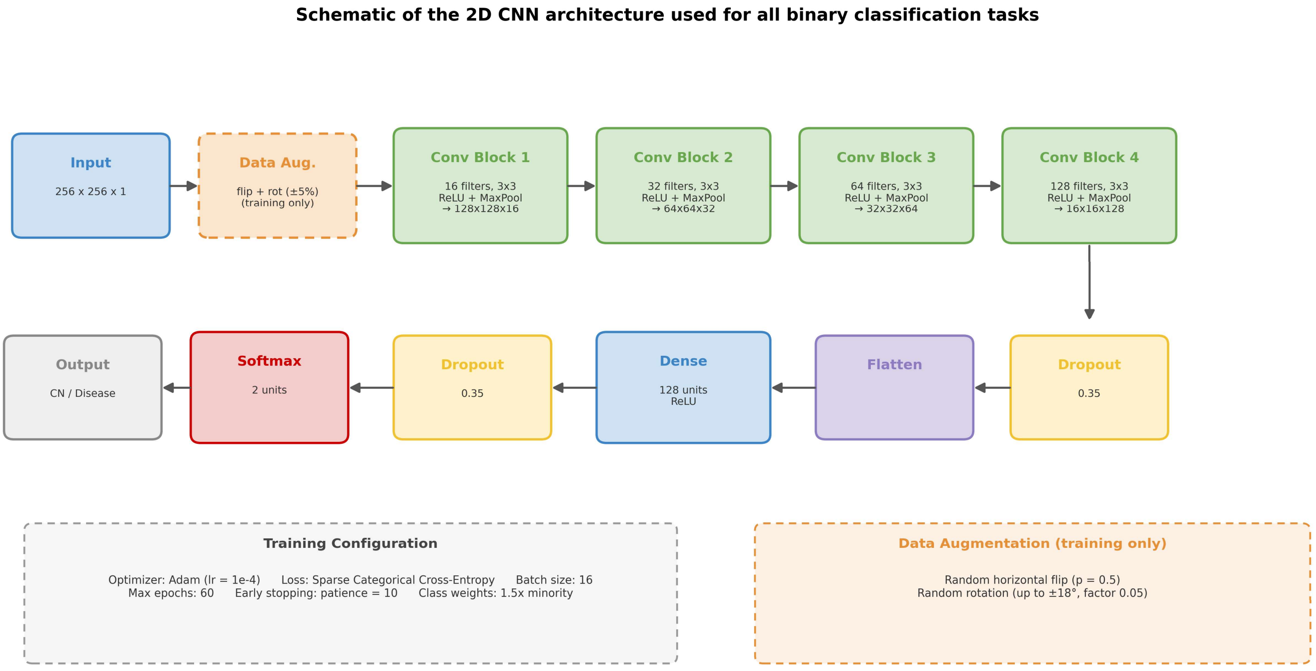

Figure S1. Schematic of the 2D CNN architecture used for all binary classification tasks. The network receives 256 × 256 × 1 grayscale axial MRI slices and passes them through four convolutional blocks (16, 32, 64, 128 filters; 3 × 3 kernels; ReLU activation; 2 × 2 max-pooling), followed by dropout (0.35), flatten, dense (128 units, ReLU), dropout (0.35), and a 2-unit softmax output layer. Online data augmentation (random horizontal flip, random rotation ±5%) was applied during training. Training used the Adam optimizer (lr = 1×10^{-4}), batch size 16, max 60 epochs, early stopping (patience 10), and class-weighted loss.

 https://doi.org/10.3390/jimaging12080352

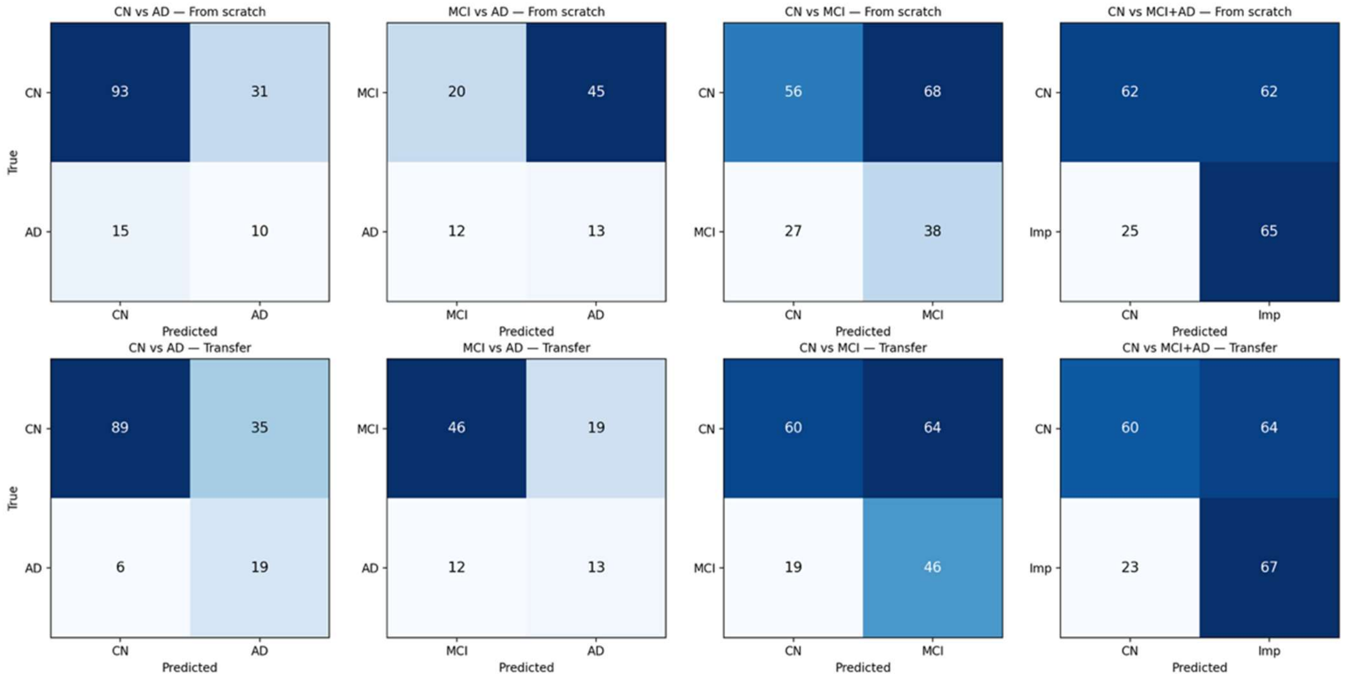

Figure S2. Subject-level confusion matrices (aggregated cross-validation) for the from-scratch (top row) and transfer (bottom row) models across the four binary tasks; cell values are subject counts. Under threshold-independent ROC-AUC the task ordering differs, with MCI vs AD the weakest transfer contrast.

https://doi.org/10.3390/jimaging12080352

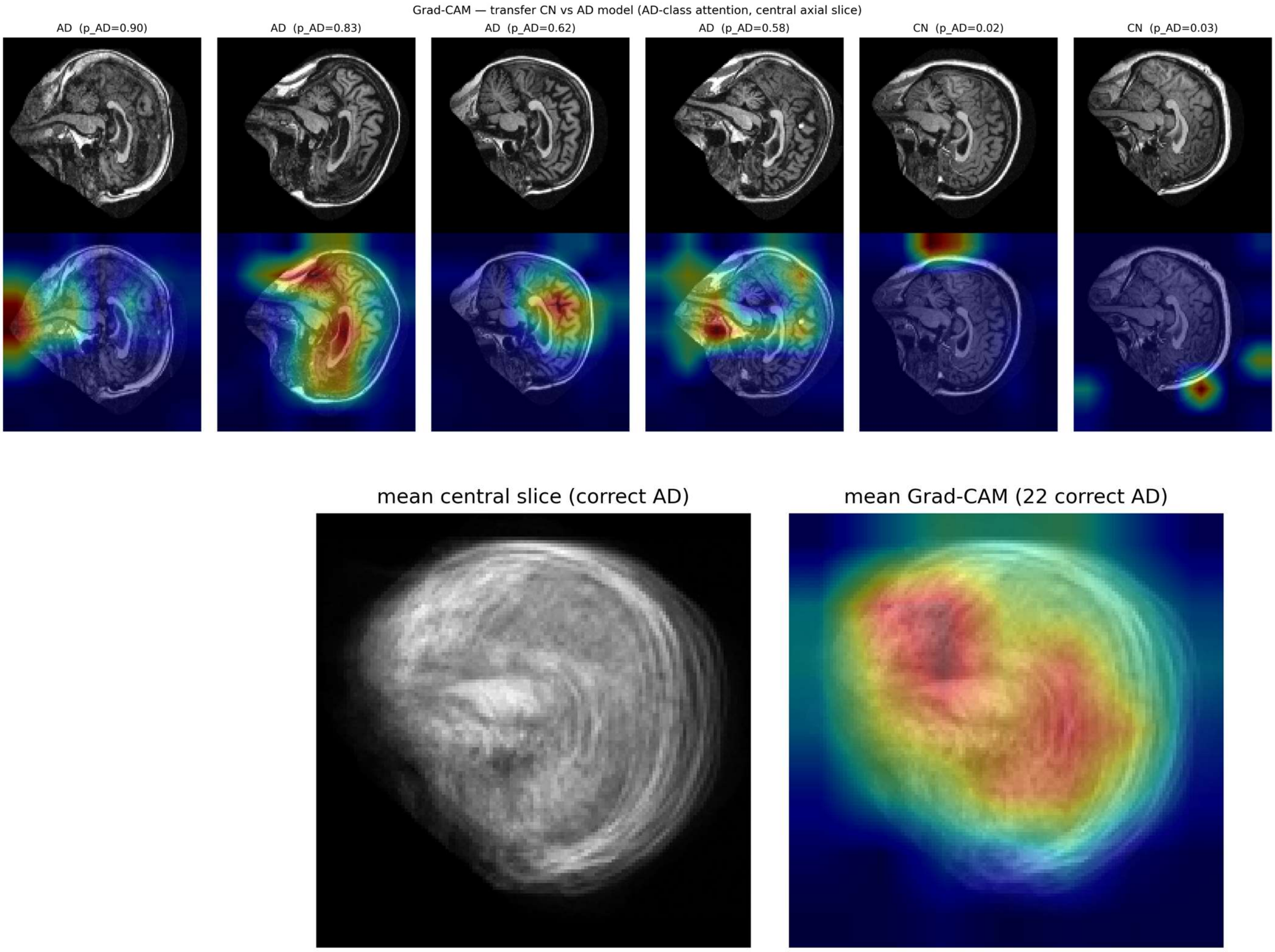

Figure S3. Grad-CAM saliency for the transfer CN vs AD model (AD-class attention on the central axial slice). Top: four correctly classified AD subjects and two correctly classified CN subjects, showing the input slice (upper row) and the Grad-CAM overlay (lower row). Bottom: mean Grad-CAM averaged over all correctly classified AD subjects. Warm regions indicate greater attention and concentrate over central/periventricular tissue in AD; the maps reflect model focus only and are not direct evidence of localized atrophy.

Table S1. Software packages and versions used in this study.

Purpose	Version	Package
Programming language	3.10	Python
Deep learning framework	2.15	TensorFlow
High-level neural network API (bundled with TensorFlow)	2.15	Keras
Numerical computing and array manipulation	1.25	NumPy
Metadata handling and tabular data processing	2.0	Pandas
Loading NIfTI-format MRI volumes	5.2	NiBabel
Image inspection and visualization	3.7	Matplotlib
Subject-level data splitting, stratified cross-validation, confusion matrix computation	1.3	Scikit-learn
Neuroimaging utilities and preprocessing	0.10	nilearn
Hierarchical clustering and dendrogram generation	1.11	SciPy

Online data augmentation pipeline (random horizontal flip, rotation, translation, zoom, contrast, and Gaussian noise)	2.15	TensorFlow / Keras (preprocessing layers)
Grad-CAM saliency computation and heatmap overlay visualization	2.15, 3.7	TensorFlow (tf.GradientTape), Matplotlib
Cloud-based development and execution environment	—	Google Colab

Disclaimer/Publisher's Note: The statements, opinions and data contained in all publications are solely those of the individual author(s) and contributor(s) and not of MDPI and/or the editor(s). MDPI and/or the editor(s) disclaim responsibility for any injury to people or property resulting from any ideas, methods, instructions or products referred to in the content.

https://doi.org/10.3390/jimaging12080352
